# Kinetics of Placental Infection by Different Smooth *Brucella* Strains in Mice

**DOI:** 10.3390/pathogens11030279

**Published:** 2022-02-22

**Authors:** Irati Poveda-Urkixo, Gustavo A. Ramírez, María-Jesús Grilló

**Affiliations:** 1 Instituto de Agrobiotecnología (IdAB, CSIC-Gobierno de Navarra), Avda. Pamplona 123, 31192 Mutilva, Spain; irati.poveda@csic.es; 2 Departamento de Sanidad Animal, Universidad de Lleida, 25198 Lleida, Spain; gustavo.ramirez@udl.cat

**Keywords:** *Brucella*, pregnant mice, placental infection kinetics, vertical transmission, cytokines

## Abstract

Abortion and reproductive failures induced by *Brucella* are the main symptoms of animal brucellosis. Laboratory animal models are essential tools of research to study the *Brucella* pathogenesis before experimentation in natural hosts. To extend the existing knowledge, we studied *B. melitensis* 16M (virulent) and Rev1 (attenuated) as well as *B. suis* bv2 infections in pregnant mice. Here, we report new information about kinetics of infection (in spleens, blood, placentas, vaginal shedding, and foetuses), serum cytokine profiles, and histopathological features in placentas and the litter throughout mice pregnancy. Both *B. melitensis* strains showed a marked placental tropism and reduced viability of pups (mainly in 16M infections), which was preceded by an intense Th1-immune response during placental development. In contrast, *B. suis* bv2 displayed lower placental tropism, mild proinflammatory immune response, and scarce bacterial transmission to the litter, thus allowing foetal viability. Overall, our studies revealed three different smooth *Brucella* patterns of placental and foetal pathogenesis in mice, providing a useful animal model for experimental brucellosis.

## 1. Introduction

Brucellosis is a widespread zoonosis caused by Gram-negative *Brucella*, facultative intracellular bacteria, which are classified in different species according to their preferred host. *B. melitensis* (small ruminants), *B. abortus* (cattle), and *B. suis* (swine) are the most pathogenic species according to their zoonotic potential and economic impact on livestock production [1]. Due to *Brucella* tropism for trophoblast cells in placenta, abortion and infertility are the most common symptoms in wildlife and domestic animals [2]. Placentitis, induced by replicative bacteria, leads to vaginal excretion and aborted foetuses, which contributes to pathogen transmission [3]. Recent reports have highlighted the importance of the metabolic pathways and nutritional conditions in the niche for *Brucella* infection [4,5]; however, the underlying mechanism of *Brucella* placental tropism remains unknown [6].

Biosafety, economical, and ethical issues in the natural host are major reasons to develop screening models in laboratory animals [7]. The mouse models of spleen infection have been extensively used to study virulence factors, vaccine attenuation, and quality control of live vaccines [7,8,9,10]. Abortion in mice is mainly manifested by reabsorption of foetuses and placentas, which is accompanied by normal delivery of viable foetuses, a fact representing a strategy of species preservation; the absence of visible signs of abortion makes interpretation difficult when it occurs, but different pregnant mouse models have been used to study *Brucella* abortion, mainly directed to study *B. abortus* infection [11,12,13,14]. Acute infection in pregnant mice induced by *B. abortus* leads to necrotising placentitis. In fact, it is suggested that abortion arises as a consequences of endoplasmic reticulum (ER) stress in trophoblast and proinflammatory events in placental tissues induced by VirB Type IV Secretion System (T4SS) of *Brucella* and its effector proteins [11,12,14,15,16]. As happens for other abortifacient pathogens [17,18,19], protective immune response against *Brucella* is mediated by a predominance of T helper type 1 (Th1) cell response characterised by secretion of IL-12 and IFN-γ [20,21]. Since normal pregnancy entails a balance of T helper cells profile to avoid foetal rejection [22,23], it was concluded that induction of a systemic Th1-response by *B. abortus* during early gestation is responsible for abortion in mice [11,12].

Although criticism has arisen around histological and physiological differences between placentas of mice, ruminants, and swine, the literature about *B. abortus* in the pregnant mouse model has evidenced how murine brucellosis resembles bovine brucellosis [6,24]. Even when *B. melitensis* and *B. suis* are the main causative agents of abortion outbreaks and economical losses, the placental pathogenesis of both smooth Brucellae has been scarcely studied in pregnant mice. Only the *B. melitensis* 16M virulent strain has been reported in this laboratory animal model [25], but the attenuated strain Rev1 is also highly abortifacient when sheep and goats are vaccinated during pregnancy, posing a high risk for human beings [9,26]. Likewise, placental infections by *B. suis* remain poorly studied. Although *B. suis* biovars 1 and 3 are the most virulent for humans, abortions by *B. suis* bv2 are of high importance and re-emergent in Europe, in farm, and wildlife animals [27]. Its non-zoonotic nature posts *B. suis* bv2 as an ideal candidate to investigate the equivalence between pregnant mice and the natural host placental infection without endangering human health.

The aim of this study was to evaluate the pathogenicity of different *B. melitensis* and *B. suis* bv2 strains in pregnant mice in order to establish the utility of a well-characterised laboratory model for pathogenic *Brucella*. The results revealed that independently of the virulence in spleens, the three smooth *Brucella* infections showed different pathogenic effects in placentas and/or litters, resembling the infection in ruminants or swine natural hosts.

## 2. Results

### 2.1. B. melitensis 16M and Rev1 Internalise More Efficiently and Induce Higher Cytotoxicity in BeWo Cells than B. suis bv2

**Figure 1:**
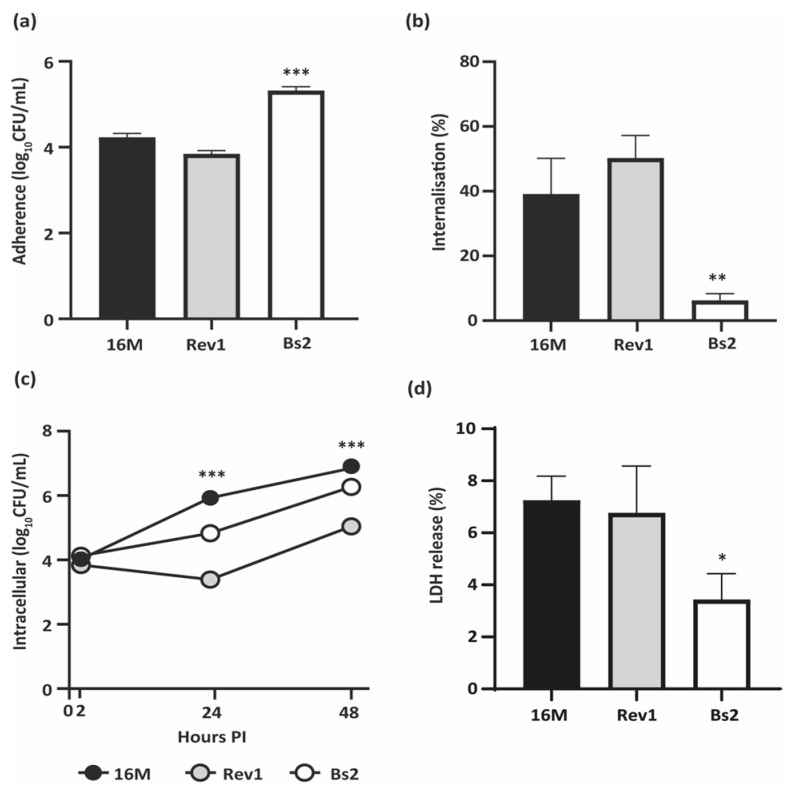
Infection of BeWo trophoblast-like cells. (**a**) Adherence and (**b**) Internalisation ratios, at 2 h post-infection (PI); (**c**) Intracellular multiplication at 2, 24, and 48 h PI; (**d**) LDH release (%) induced at 48 h PI. BeWo cells were infected by *B. melitensis* 16M or Rev1 or by *B. suis* bv2 (Bs2) at an MOI 1:100 and lysed at selected intervals for CFU counting and supernatant harvesting. Each study was performed in three independent experiments and each datum was obtained in triplicate; results represent the mean ± SD (*n* = 3) of one representative experiment. PLSD test: * *p* ≤ 0.05, ** *p* ≤ 0.01, *** *p* ≤ 0.001 vs. other strains of the same experiment.

First, we evaluated the ability of *B. melitensis* 16M, Rev1, and *B. suis* bv2 to infect BeWo human trophoblast-like cells. In comparison to both *B. melitensis* strains, *B. suis* bv2 showed high adherence and minimal internalisation in BeWo cells but drawing an active intracellular replication pattern (Figure 1a–c). Rev1 showed a faint reduction in its counts at 24 h post-infection (PI) followed by an active replication at 48 h PI, but reaching lower levels of infection than those of the virulent strains (Figure 1c).

Then, we measured the cytotoxicity induced by each strain in infected trophoblasts by measuring the levels of lactate dehydrogenase (LDH) released. *B. melitensis* virulent and attenuated strains displayed similar cytotoxicity between them and significantly higher (*p* ≤ 0.05) than that displayed by *B. suis* bv2 (Figure 1d).

### 2.2. B. melitensis 16M and Rev1 Exhibit More Tropism for Mouse Placentas Than B. suis bv2

In the screening experiment, the three experimental groups showed similar levels of spleen infection, but both *B. melitensis* strains induced a severe splenomegaly that was significantly higher than that induced by *B. suis* bv2 (Table 1). In mature placentas and foetuses after 18.5 days of gestation (DG), 16M virulent and Rev1-attenuated strains showed similar multiplication rates, being both higher than those observed in spleens (*p* ≤ 0.005). In contrast, *B. suis* bv2 showed less marked tropism for placentas and minimal vertical transmission to foetuses, despite the spleen infections being higher than those observed in *B. melitensis* mice.

**Figure 2:**
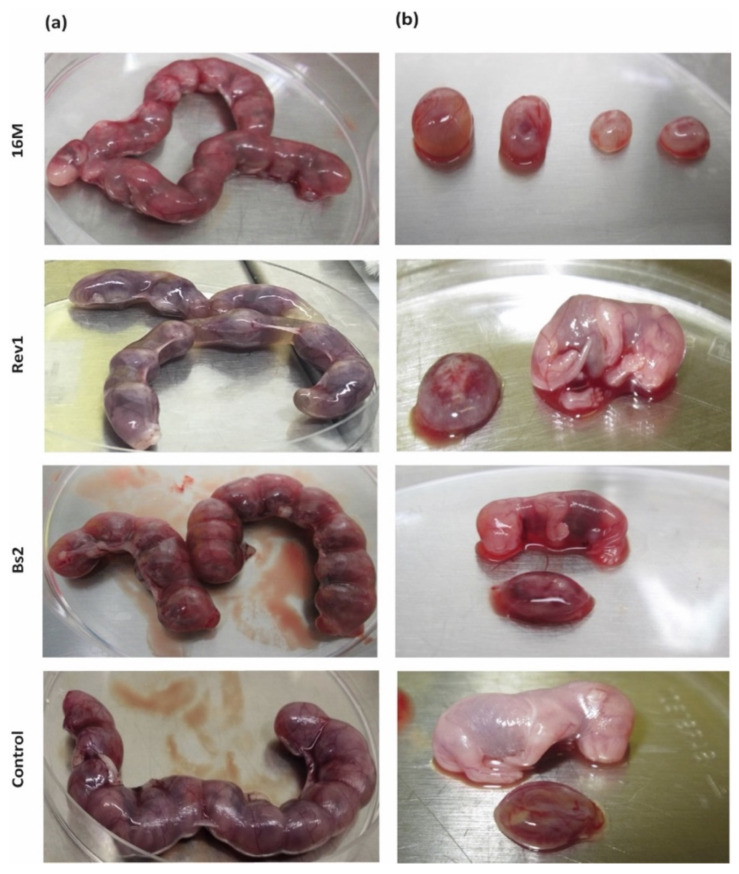
Macroscopic features of (**a**) gravid uterus and (**b**) placental discs and foetuses at 18.5 DG. Mice were inoculated and necropsied as described in the footnote of Table 1.

Despite the high rates of *B. melitensis* infection in foetuses (6–7 log CFU/g), macroscopic injuries were observed mainly in the 16M-infected ones, showing foetuses very small and with different degrees of reabsorption (Figure 2b). In contrast, Rev1 allowed normal body development of foetuses, showing in some of them, a distension of the uterine lumen with serous fluid between contiguous conceptus (Figure 2a). The macroscopic aspect of *B. suis* bv2 gravid uterus and foetuses did not show specific lesions, as well as the PBS control group did.

### 2.3. Kinetics of B. Melitensis and B. suis bv2 Infections in Spleens, Blood, Placentas and Vaginal Shedding and Cytokines Triggered by Infection, along Pregnancy in Mice

#### 2.3.1. *B. melitensis* 16M and Rev1 Induce Higher Splenomegaly than *B. suis* bv2 in Pregnant Mice, Associated to Granulomas

**Figure 3:**
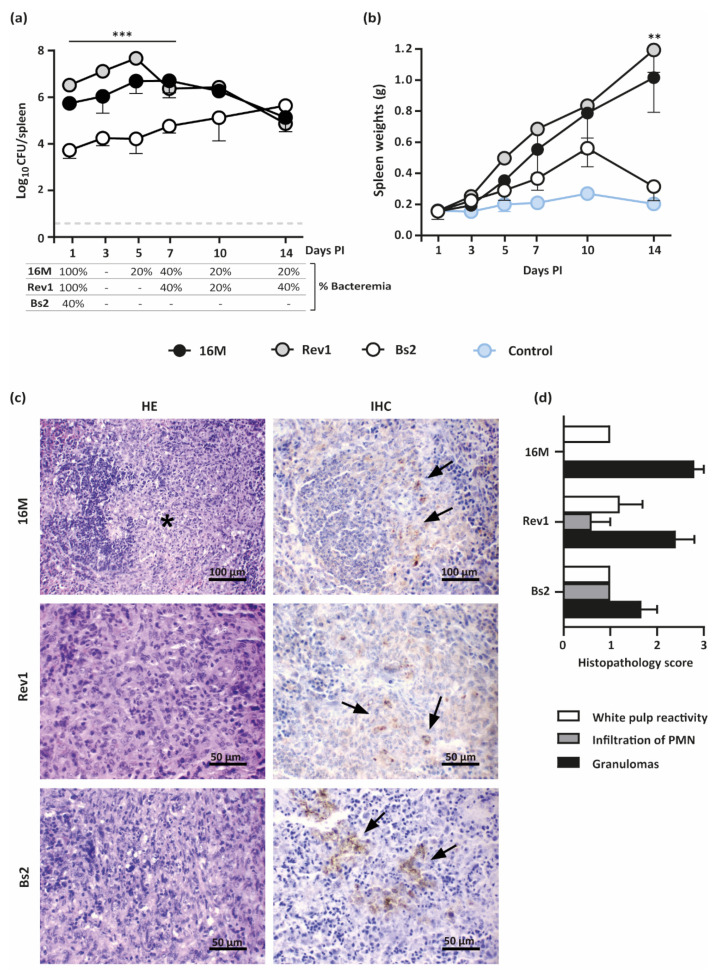
Kinetics of spleen infections and splenomegaly in pregnant mice infected by *B. melitensis* or *B. suis* bv2 strains. (**a**) Kinetics of spleen infections (mean ± SD) and bacteraemia (percentage of positive animals); (**b**) Kinetics of spleen weights (mean ± SD); (**c**) Representative images of HE (**left** column) and IHC (**right** column) of spleens infected with *B. melitensis* 16M or Rev1 or by *B. suis* bv2 (Bs2), at 18.5 DG. The asterisk indicates epithelioid macrophages infiltration within the marginal zone and lymphoid follicles; arrows indicate *Brucella* antigen DAB stained within macrophages cytoplasm.; (**d**) Histopathological score of spleen injuries severity (0–3) at 18.5 DG, based on intensity and distribution of granulomas, PMN, and white pulp reactivity (median ± SE). Groups of 30 pregnant CD1 mice were intraperitoneally inoculated at 4.5 DG with 6–7 × 10^5^ CFU/mouse or PBS control and then necropsied and sampled (*n* = 5) at selected intervals. Detection limit: 3.3 CFU/spleen = 0.52 logs. Fisher’s PLSD test: ** *p* ≤ 0.01 or *** *p* ≤ 0.001 for *B. melitensis* strains vs. *B. suis* bv2 at a given point in time.

The kinetics of the bacterial loads in spleens and splenomegaly throughout pregnancy is presented in Figure 3a,b. Both *B. melitensis* strains showed similar patterns of infection along pregnancy, with high levels of colonisation during the first 10 days PI that declined thereafter; the splenomegaly increased throughout the experiment, reaching the maximum at 18.5 DG. In contrast, *B. suis* bv2 induced lower levels of spleen infections than *B. melitensis* during 7–10 days PI, peaking thereafter; the spleen weights of these animals increased moderately and progressively until reaching a peak at 10 days PI, dropping thereafter to the basal levels of the PBS control. PBS inoculated females also showed a peak of physiological splenomegaly at 10 DG (0.27 g/spleen); nevertheless, splenomegaly induced by *B. melitensis* 16M and Rev1 exceeded six times these values at late gestation.

Histopathologic changes by haematoxylin–eosin (HE) in all infected spleens were characterised by aggregates of epithelioid macrophages with few scattered polymorphonuclear neutrophils (PMN) in the marginal zone and lymphoid follicles infiltration (Figure 3c,d). The space occupied by these histiocytic infiltrates was significantly higher in both *B. melitensis* groups than in the *B. suis* bv2 group, but the histiocytic infiltrates were more PMN-rich (pyogranulomatous) in the latter. Mild lymphoid hyperplasia in the splenic white pulp was noted for all groups. Brucella antigen was immunohistochemically (IHC) detected within the cytoplasm of epithelioid macrophages and blood sinusoids in the splenic red pulp for all infected groups (Figure 3c,d).

To delve into the spread of infection, bacteraemia was assessed at each interval PI. Both *B. melitensis* strains were detected at 1 day PI in all pregnant mice but only in two out of five infected by *B. suis* bv2 (Figure 3a), showing levels below 5 CFU/100 µL of blood (not shown). Successive intervals revealed intermittent periods of bacteraemia by *B. melitensis* at levels below 5 CFU/100 µL and non-detectable *B. suis* bv2.

#### 2.3.2. *B. melitensis* Strains but Not *B. suis* bv2 Display a Th-1 Mediated Immune Response in Pregnant Mice

**Figure 4:**
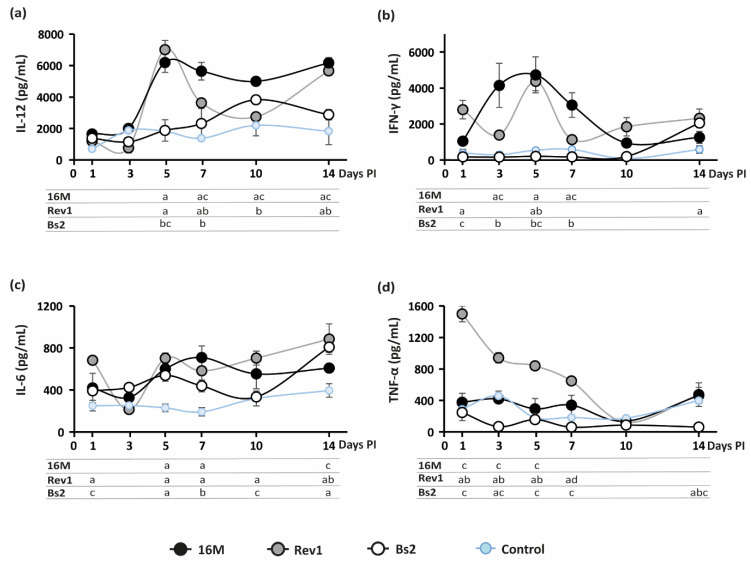
Kinetics of serum cytokine levels along pregnancy in mice. (**a**) IL-12; (**b**) IFN-γ; (**c**) IL-6; (**d**) TNF-α. Mice were inoculated and sampled at selected intervals, as detailed in the footnote of Figure 3; cytokine levels (pg/mL) were measured by ELISA (mean ± SE) in blood serum. Sidak’s multiple-comparison test: *p* ≤ 0.05, ^a^ vs. PBS control, ^b^ vs. 16M, ^c^ vs. Rev1.

Maternal immunity modulation seems essential for pregnancy maintenance [28,29]. In particular, an appropriate shift of the immunological balance toward antibody-mediated T-helper 2 (Th2) allows a successful pregnancy. By contrast, cell-mediated Th1 immune response has had a detrimental impact on the continuity of the gestation [30,31]. Virulent B. abortus infection is known to trigger a predominant Th1 cytokine response mainly mediated by IFN-γ, which contributes to abortion in mice [11]. Therefore, we examined the kinetics of IL-12, IFN-γ, IL-6, and TNF-α cytokines induced by 16M, Rev1, and *B. suis* bv2 strains in blood sera, along pregnancy. As shown in Figure 4, mice infected with *B. melitensis* 16M or Rev1 displayed a significative increase in IL-12 and IFN-γ at 5 days PI, decreasing thereafter, more markedly in Rev1; IL-12 peaked again at the end of pregnancy, overlapping the highest peak of splenomegaly (Figure 3b).

Moreover, Rev1 triggered a high release of TNF-α during the first week PI. In contrast to *B. melitensis*, *B. suis* bv2 followed the pattern of the PBS group, except for a peak of IL-6 at 5 and 7 days PI; and a peak of IL-12 at 10 days PI; the latter was coincident with a physiological increase in this cytokine in the PBS control as well as of the spleen weights (Figure 3b). No significative IL-12, IFN-γ, and TNF-α fluctuations were detected for *B. suis* bv2 and PBS control groups along pregnancy. IL-6 is considered a key mediator of proinflammatory response to LPS [32,33,34]; its levels rose significantly at 5 and 7 days PI in all Brucella infected mice vs. the PBS control group.

#### 2.3.3. Placental Infections Increase Progressively throughout Pregnancy, More Markedly in *B. melitensis* than That of *B. suis* bv2 

**Figure 5:**
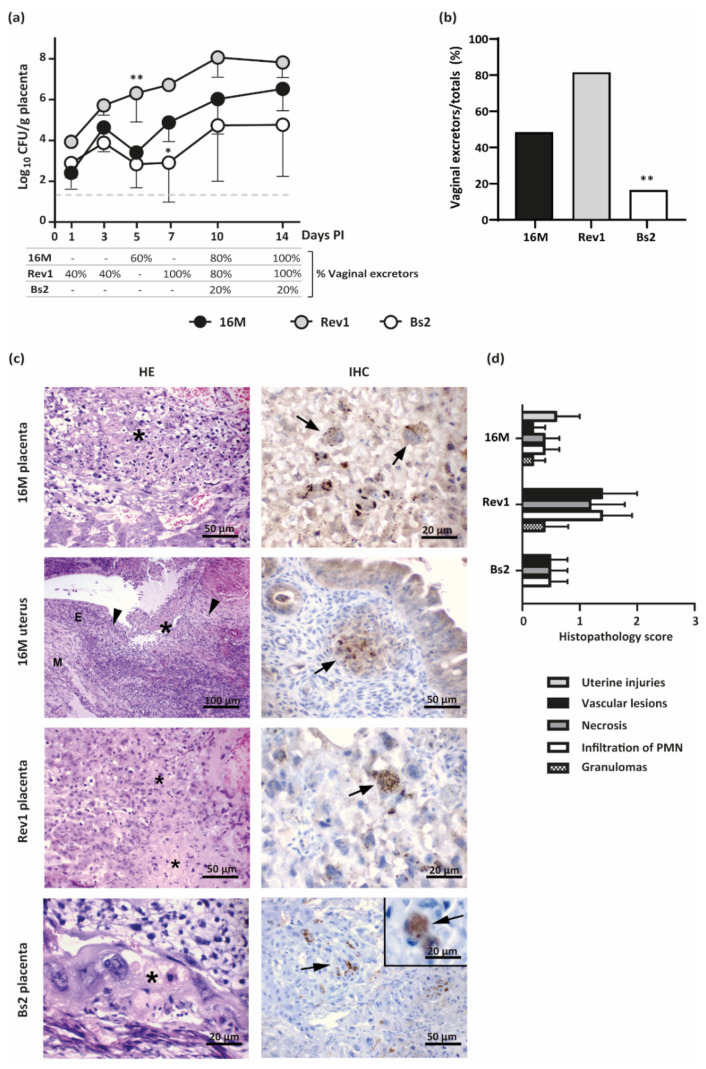
Kinetics of placental infections and vaginal shedding along pregnancy and histopathological findings at 18.5 DG in mice infected by *B. melitensis* or *B. suis* bv2. (**a**) Placental infections (mean ± SD) and percentage of vaginal shedding along pregnancy; (**b**) Percentage of *Brucella* excretors by vaginal vs. the total pregnant mice of the study; (**c**) Representative images of the HE (**left** column) and IHC (**right** column) in placentas or uterus at 18.5 DG. Asterisks indicate foci of necrosis with degenerated PMN and karyorrhectic debris; arrowheads indicate uterine lesions with PMN or granulomatous infiltration; M = myometrium; E = endometrium; Arrows: *Brucella* antigen in TGC or in uterine epithelioid macrophages; (**d**) Histopathological score injuries (severity 0–3) at 18.5 DG, based on intensity and distribution of granulomas, PMN, necrosis, and vascular lesions (median ± SE). Pregnant mice were inoculated and sampled as detailed in the footnote of Figure 3. Fisher’s PLSD and Chi-square tests: * *p* ≤ 0.05 or ** *p* ≤ 0.01 vs. other groups of the point time.

As shown in Figure 5a, the three *Brucella* strains showed an active multiplication in placental tissues throughout pregnancy, which were more marked for both *B. melitensis* strains. Interestingly, Rev1 colonised the placental tissues at higher levels than 16M, which was probably due to the lower levels of damage of the placental tissues produced by the former. In contrast to Rev1, 16M and *B. suis* bv2 suffered a transitory reduction in bacterial counts at 5 days PI; afterwards, 16M regained its infective potential until the end of the experiment, while *B. suis* bv2 showed lower rates of placental colonisation. In contrast to both *B. melitensis* strains, *B. suis* bv2 showed higher bacterial loads in spleens than in placentas.

According to placental infections, Rev1 was detected earlier and in a higher percentage of the mice vaginas than 16M during the first 7 days PI; thereafter, Rev1 maintained its high levels of excretion, while 16M shedding increased progressively. Both *B. melitensis* strains reached 100% of excretors at 14 days PI (Figure 5a). In contrast, *B. suis* bv2 was only found in 20% of mice vaginas at the latest stages of the pregnancy. Considering all data together, all mice shedding the pathogen through the vagina showed placental infection as well. Thus, Rev1 and 16M were shed (80% and 47.4%, respectively) in a higher proportion of pregnant animals than *B. suis* bv2 (15.8%) (Figure 4b).

Microscopically, *B. melitensis* 16M and Rev1 induced placental lesions more severe and extensive than those induced by *B. suis* bv2. Foci of necrosis with degenerated PMN, and karyorrhectic debris were observed in all infected groups with less extension in mice infected with *B. suis* bv2. In mice infected by *B. melitensis*, the trophoblast giant cells (TGC) and spongiotrophoblasts were infiltrated by PMN, showing coagulative necrosis multifocal to coalescing (Figure 5c,d). In addition, these animals evidenced microthrombi in blood vessels of the junctional zone and labyrinth, especially in the Rev1 group. The uteruses infected by *B. melitensis* 16M carrying all foetuses reabsorbed showed PMN or pyogranulomatous infiltration, necrosis, haemorrhage and/or thrombosis. In contrast, no lesions were observed in the uterine wall of *B. suis* bv2 infected mice.

Immunostaining of infected placentas revealed that bacteria were present in TGC and blood vessels in the placental labyrinth (Figure 5c). The cytoplasm of the infected TGC contained variable amounts of positive staining material surrounding cell nuclei. Furthermore, some intact TGC exhibited brown staining within the cytoplasm, indicating the presence of intracellular *Brucella*. No immune-positive signal was detected in the control group.

#### 2.3.4. *B. melitensis* 16M and Rev1 but Scarcely *B. suis* bv2 Are Vertically Transmitted and the Infections Affect Foetal Viability 

Foetal viability was evaluated at late pregnancy, since at earlier stages, the small size of foetuses thwarted an appropriate dissection from placental envelops. The litter size was not affected by *Brucella* infection, but foetal viability was significantly reduced (*p* ≤ 0.001) in 16M and Rev1 groups (5/5 and 2/5 females with non-viable pups, respectively). In contrast, *B. suis* bv2 and PBS control groups showed most of the foetuses viable without significant differences in litter weight, foetal infections, and viability (Table 2).

## 3. Discussion

Abortion is the main symptom of *Brucella* infections. The mechanism underlying the placental tropism is not completely understood; experimental animal models can be a useful tool to extend the understanding. In this work, we studied for the first time the ability of the smooth *B. melitensis* Rev1 and *B. suis* bv2 strains to infect BeWo trophoblast-like cells as well as mice placentas and foetuses. Interestingly, Rev1 was highly pathogenic in pregnant mice but allowed more viability of the litter than 16M, as reported for small ruminants [26]. Although both *B. melitensis* strains showed different patterns of intracellular multiplication in BeWo, no cytotoxicity differences were detected between them. In contrast to previous reports [35,36], we were able to detect higher LDH concentration in infected than in non-infected BeWo cells, which is in agreement to those detected by Salcedo and cols. in 16M infected JEG-3 trophoblast cells [16]. The similar cytotoxicity evidenced by *B. melitensis* virulent and attenuated strains seemed to correlate to the in vivo placental tropism and damage induced by both strains [37,38]. *B. suis* bv2 exhibited reduced efficacy of internalisation but active intracellular multiplication, in contrast to both *B. melitensis* and to *B. suis* bv1 1330 [16]. While *Brucella* internalisation has been widely studied in macrophages and epithelial cells [39,40,41], adhesion and the internalisation mechanism in trophoblast cells requires further clarification. Recently, two proteins of *B. suis* bv1 1330 (BmaA and BmaB) have been described as feasible adhesins involved in trophoblast adhesion process [42]. Although *Brucella* strains share a high degree of genome similarity [43], *bmaA and bmaB* loci seem to correspond to pseudogenes in *B. melitensis* [42]. Accordingly, variations in the functional adhesins between *Brucella* species have been related with host preferences [44]; this fact could explain different adhesion and internalisation ratios between *B. melitensis* and *B. suis* bv2 observed in this study. Although *B. suis* bv2 replicates more efficiently than Rev1, the cytotoxicity induced was lesser than those expected for a natural virulent strain.

As in our work, trophoblastic cell lines have demonstrated their suitability for *Brucella* pathogenesis research [15,16,33,34,36,43]. This finding could be attributable to the presence of detectable concentrations of hypothetical factors of *Brucella* tropism, such as erythritol and aldose reductase [4,45]. Nevertheless, pregnancy involves a complex and dynamic physiological status only evaluable in animals. Thus, the pregnant mice models represent an attractive tool to investigate abortions in mammals by *Brucella* [6].

Whereas mice show an apparent resistance to abortion by *Brucella* spp. [7], previous research has determined the existence of a narrow window at mid pregnancy in which *Brucella* is able to effectively colonise the placenta [46]. More recently, Kim et al. established a pregnant mouse model with the highest abortion rate by infecting animals at 4.5 DG. This experimental model has been subsequently used to increase understanding in *Brucella* placental pathogenesis [12,13,14,15,47], and it was used in the present study to describe the behaviour of classical strains such as *B. melitensis* 16M, Rev1, and *B. suis* bv2. As with *B. abortus* 2308 [11,24], the three studied strains were able to infect placentas at 18.5 DG. Marked tropism of 16M and Rev1 was underlined according to higher bacterial isolation in placentas and by IHC analysis in comparison with splenic tissues. In contrast, *B. suis* bv2, which has a different abortifacient behaviour in its natural host [9], showed a moderate degree of placental colonisation in line with splenic infection. This moderate tropism for murine placentas could be related to its host specificity [48]. In addition, *B. suis* bv2 infection does not always induce abortion in swine but rather variable severity of placentitis [27].

Kim et al. have reported that *B. abortus* induces late-pregnancy abortion in mice [11], resembling bovine infections [9,49]. However, this pregnant mouse model can limit the understanding of swine brucellosis, since abortion in sows can occur at any stage of pregnancy [27]. Furthermore, *Brucella* infection involves a dynamic process throughout pregnancy; thus, we used a model to study the infection for the full duration of pregnancy.

*Brucella* spleen infections observed in pregnant mice did not differ from that reported in non-pregnant mice during the first 2 weeks PI [50,51,52,53]. However, all infected mice showed higher splenomegaly than that expected at 14.5 DG as a physiological expansion of the red pulp [54]. In 16M and Rev1 groups, the splenomegaly was also accompanied by granulomas, leading to a ten-fold increase in the spleen weight at the end of pregnancy. These peaks of splenomegaly are accompanied by an increase in circulating IL-12 at 14.5 DG for *B. suis* bv2 and at 18.5 DG for *B. melitensis*.

It seems likely that an early inoculation during embryo implantation (4.5 DG) period [55,56] allows an effective colonisation of placental envelops, showing a progressive multiplication of 16M and Rev1, peaking at the end of gestation. It has been reported that *Brucella* localises preferentially in TGC [11,25], which are mainly placed at the junction and labyrinth layers [57]. Our results agree with these previous findings, since *Brucella* antigens were immunohistochemically detected in the TGC in all the infected groups under study. In mice, TGC are one of the major types of placental endocrine cells, playing a crucial role for conceptus implantation/evolution, embryonic cavity remodelling, and promotion of maternal blood flow [57,58]. It has been proposed that TGC could be hijacked by *Brucella* so that cell functions could not be exhibited completely, thus leading to abortion due to inhibition of implantation and placental development [11].

The TGC lineages arise between 5.5 and 8.5DG, first to mediate implantation and uterus invasion and subsequently to produce hormones, cytokines, and growth factors that contribute to gestation maintenance [57,58]. The proliferation of TGC during these first stages of placental development seems to favour *Brucella* multiplication. Interestingly, 16M and *B. suis* bv2 counts decreased at 9.5 DG (5 days PI), the stage at which maternal blood enters the labyrinth [56]. This phenomenon could be explained by the increased irrigation of placental tissues facilitating cell-mediated immune response, which is followed by the marked cytokine release at 5 days PI.

Whereas cytokines levels in *B. suis* bv2 and PBS groups did not show marked dysregulation, *B. melitensis* strains induced a significant release of Th1 cytokines at 5 days PI by a synergic effect of IL-12 and IFN-γ [7]. Analogous cell-immune response was also evidenced in pregnant ruminants inoculated with both 16M and Rev1 strains [59,60]. IFN-γ is essential to control *Brucella* infections [61], showing a transient peak that has been related to promote abortion in pregnant mice infected by *B. abortus* [11]. Likewise, abortion and reduced foetal viability caused by 16M and Rev1 infections may be promoted by this transient IFN-γ peak at 9.5 DG, which is delayed with respect to those reported for *B. abortus*.

Nevertheless, the stealthy behaviour of virulent *B. melitensis* 16M and *B. suis* bv2 strains was evidenced by minimum TNF-α serological levels throughout pregnancy. This result is not in contradiction with those described for *B. abortus* [11] and those reported in non-pregnant mouse and macrophages [62,63,64]. In contrast, the early secretion of TNF-α by Rev1 vaccine may explain a significant reduction in litter size by hampering embryo implantation and subsequent development [65,66].

Placenta constitutes a selective barrier for pathogens and maternal immune rejection of the allogenic conceptus [67]. Accordingly, Wang et al. reported that 16M placental infection precedes foetal infection, but when placenta is already infected, vertical transmission cannot be prevented [25]. Our results suggested that foetal infection occurs when placental counts exceed a certain threshold. In fact, *B. melitensis* in mice can be correlated with those of placental infections and abortions small ruminants [59,60,68]. Viability of the pups correlated with infection in the reproductive tract, since foetuses from *B. suis* bv2-infected dams presented low mortality, with no apparent macroscopic injuries.

To our knowledge, this is the first kinetics study of *Brucella* infections in placentas, vaginal shedding, and litters throughout pregnancy in mice, in contrast to other experimental designs [11,45,65]. The experimental conditions and results obtained in pregnant mice cannot be directly extrapolated to natural hosts of different age, sex, body weight, and physiological and reproductive status due to obvious histological and physiological placental divergences [67,68]. However, a well-standardised mouse model represents a useful tool to select further study of *Brucella* placental pathogenesis and abortion, as well as a screening to select safer vaccine candidates.

## 4. Materials and Methods

### 4.1. Bacterial Strains, Media, and Culture Conditions

The bacterial strains used in this study were *B. melitensis* bv1 16M (virulent) and Rev1 (attenuated), and *B. suis* bv2 (strain 198 Thomsen-like; Bs2 [69]). All strains were stored at −20 °C in 10% skimmed milk supplemented with 3% lactose (both PanReac AppliChem). For cellular infections, *Brucella* was cultured overnight on Trypticase Soy Broth (TSB, Pronadisa, Madrid, Spain) at 37 °C under shaking (150 rpm). For mouse infections, *Brucella* was cultured on Blood Agar Base No.2 (BAB, Oxoid, Hampshire, UK) for 48 h, resuspended in sterile phosphate saline solution (PBS, pH 7.2), and adjusted to OD_600 nm_ = 0.170 absorbance (Abs) by a SmartSpec Plus Spectrophotometer (Bio-Rad, Madrid, Spain). The inocula were adjusted by dilutions in PBS, and the exact number of Colony-Forming Units (CFU) was assessed retrospectively by serial ten-fold dilutions in PBS and plating by triplicate in BAB (37 °C, 5 days), as described elsewhere [70]. All *Brucella* manipulations were performed at the registered BSL3 facilities (code A/ES/15/I-05) of the Instituto de Agrobiotecnología.

### 4.2. BeWo Infections

BeWo choriocarcinoma immortalised human trophoblast cells [71] (ATCC CCl-98; Sigma-Aldrich, Madrid, Spain) were cultured in F-12K medium (Kaighn’s modification) supplemented with 10% of inactivated foetal bovine serum (FBS) and 2 mM L-glutamine (GlutaMAX^TM^ 100X Gibco) at 37 °C in a 5% CO_2_ atmosphere. Trophoblastic cells (5 × 10^4^ cells/well in 24-well plates) were infected, by triplicate, at a multiplicity of infection (MOI) of 100, centrifuged at 400× *g* for 5 min, and incubated for 30 min (37 °C, 5% CO_2_), as reported [16]. Cells were extensively washed, exposed to 50 µg/mL of gentamycin (Gm_50_) for 1.5 h, and lysed with 0.1% Triton 100× (Sigma-Aldrich) in PBS. The number of CFU/mL was determined in both Gm_50_-treated (intracellular bacteria) and untreated (total bacteria) wells by serial dilutions and plating on BAB (37 °C, 5 days). At this point in time (2 h post-infection; PI), bacterial adhesion was calculated as mean ± SD of individual log_10_ (total CFU/mL—intracellular CFU/mL) [40], and the internalisation ratio was calculated as (intracellular CFU: total CFU) × 100. To monitor bacterial intracellular survival, the log_10_ CFU/mL from Gm_50_-treated wells was determined at 2, 24, and 48 h PI.

The concentration of lactate dehydrogenase (LDH) released was measured at 2, 24, and 48 h PI by the commercial kit CytoTox96 Non-radioactive, Cytotoxicity Assay (Promega, Madrid, Spain) following the manufacturer’s instructions. Briefly, supernatants were collected and mixed with the kit reagent and incubated for 45 min. The reaction was stopped, and the Abs was read at OD_492nm_ (Multiskan Ex, Labsystems). The maximum lysis control (MLC) and the background control (BC) of each plate were determined by OD_492nm_ Abs readings of wells containing the reagent incubated with either non-infected cells (for MLC) or without cells (for BC). The percentage of cytotoxicity was calculated as follows: [(Abs. infected cells—Abs. BC)/(Abs. MLC—Abs. BC)] × 100.

### 4.3. Mice Experiments

#### 4.3.1. Ethics and Animal Welfare Statements

Experiments with mice were carried out in strict accordance with the recommended guidelines of Federation of European Laboratories of Animals Science Associations (FELASA) and Animal Research Reporting of in vivo Experiments (ARRIVE). Experimental procedures were based on brucellosis standard procedures [7], performed by accredited personnel, and authorised by the competent authority (code PI-025-14 of the Navarra government) in compliance with the European and Spanish legislation on the use of animals for experimentation and other scientific purposes (RD 53/2013, Order ECC/566/2015 and Directive UE 2010/63). No humanitarian endpoints were necessary, since bacterial inoculations did not induce any clinical nor signs of suffering, and all samples were taken under anaesthesia or killed before anaesthetic recovery.

#### 4.3.2. Animals and Biosafety

Animals were purchased from Charles River (Elbeuf, France) and acclimatised for 2 weeks in the authorised IdAB animal facilities (code ES/31-2016-000002-CR-SU-US) in microisolator cages with water and food ad libitum. Pregnant mice were moved to the IdAB registered BSL3 facilities (code A/ES/15/I-05) to perform the *Brucella* inoculations, sampling, and handling until the end of the experiment.

#### 4.3.3. Pregnancies Synchronisation and Experimental Infections of Pregnant Mice

Eight-week-old CD1 female mice were naturally mated by introducing one male with 5 females in a cage for 2 days. Pregnancies were confirmed by the presence of a vaginal plug, and at 4.5 ± 1 DG, groups of pregnant mice were intraperitoneally inoculated with 0.1 mL of inoculum containing 6–7 × 10^5^ CFU of 16M, Rev1 or *B. suis* bv2, or PBS (control group). Animals were euthanised by cervical dislocation at indicated intervals.

#### 4.3.4. Screening of Infection in Pregnant Mice

A preliminary experiment was performed in groups of 5 inoculated pregnant mice (see Section 4.3.3) and sampled at 18.5 DG by aseptically dissection of spleens, placentas, and foetuses. The number of viable foetuses and log_10_ CFU/g of each organ was determined by homogenisation in PBS and plating in BAB (37 °C, 5 days), as detailed elsewhere [70].

#### 4.3.5. Kinetics of Infection during Pregnancy

Groups of 30 inoculated pregnant mice (see Section 4.3.3) were sampled (*n* = 5) at 1, 3, 5, 7, 10, and 14 days PI by aseptically collection of blood, vaginal fluid, spleens, placentas, and foetuses, and processed as follows:

(i) Blood samples were obtained by retroorbital sinus puncture under anaesthesia and used for bacteraemia detection and for cytokine analysis in serum. For bacteraemia, blood samples were collected with 10% of 50 mM EDTA (Sigma-Aldrich), diluted in PBS-0.1% Triton-100× (Sigma-Aldrich) as described elsewhere [72], and 100 µL were plated in BAB and cultured (37 °C, 5 days).
(ii) Vaginal fluid was collected individually by vaginal washes with 100 µL of sterile PBS previous external decontamination of vulva with povidone–iodine 10% (Betadine^®^). Vaginal excretion of *Brucella* was determined by plating of collected fluid in CITA selective medium [73].
(iii) At necropsy, spleens were aseptically removed, individually weighted, and processed. Placental discs were detached from uterine walls and considered as a pool of each dam. When possible, the foetus was dissected from placental envelops tissue. The pool of pups was externally washed with ethanol (70%) and air dried in order to avoid overestimation of foetal infection from amniotic fluid. Each organ or pool was homogenised in sterile PBS, and ten-fold dilutions were plated in BAB to determine viable bacterial counts as previously described [70].

Foetal viability was evaluated by presence/absence of foetal movement, skin colour, and foetal size and development. Percentage of viability was calculated using the following formula: (number of viable pups per litter/total number of pups per litter) × 100.

### 4.4. Serum Cytokine Analysis 

Individual serum samples were obtained by centrifugation (1000× *g*, 10 min) of coagulated blood. Serum levels of interleukin 6 (IL-6), 12p40 (IL-12), interferon gamma (IFN-γ), and tumour necrosis factor alpha (TNF-α) cytokines were measured using a commercial Enzyme-Linked Immunosorbent Assay (ELISA, BD OptEIA^TM^), following the manufacturer’s instructions.

### 4.5. Histopathological Analysis

Placental disc and spleen samples were fixed in 10% neutral buffered formaldehyde (PanReac AppliChem) and processed as routinely for embedding in paraffin wax, cutting in 4 µm sections, and staining with haematoxylin–eosin (HE). Histopathological analysis was performed by double blinding following the described criteria [14]. A histopathological score from 0 to 3 was given to each spleen and placenta according to the intensity and distribution of granulomas, PMN, necrosis foci, and vascular lesions (0: absence; 1: mild focal; 2: moderate or multifocal; 3: severe multifocal to diffuse).

Similar sections (3 µm) of placenta and spleen samples were used for IHC analyses. Samples infected with *B. melitensis* were incubated with polyclonal anti-*Brucella* serum (S-LPS) raised in sheep (1:1000). After incubation with secondary antibody (anti-sheep IgG- biotin-labelled, the (Thermo-Fisher, Waltham, MA, USA ) reaction was visualised using the avidin–biotin–peroxidase complex technique with 3,3′-diaminobenzidine tetrahydrochloride (DAB; Sigma-Aldrich, St. Louis, MO, USA). *B. suis*-infected samples were incubated with polyclonal A-epitopes specific serum raised in rabbit (1:1000), and reaction was visualised with an EnVisionTM, FLEX, kit (Agilent Technologies-Dako, Santa Clara, CA, USA) using diaminobenzidine chromogen as a substrate. Sections were lightly counterstained with Harris’ haematoxylin. Non-infected murine placental sections were used as negative controls.

### 4.6. Statistical Analysis

The statistical analysis was performed using GraphPad Prism^®^ 8.3.0. Software (San Diego, CA, USA). Analysis of CFU data was performed using one- or two-way analysis of variance (ANOVA) followed by the indicated multiple comparison test in each case. Variations in serological cytokine levels from sera were analysed by ANOVA and Sidak’s multiple comparison tests. In all cases, statistical significance was established in *p* ≤ 0.05.

## Figures and Tables

**Table 1:** Screening of *Brucella* spp. infection in pregnant mice at 18.5 DG.

Group	Spleen		Reproductive Tract			
Strain	Weight (g)	Infection (log_10_ CFU/g)	Placentas		Foetuses	
			Females Infected/Total	Infection (log_10_ CFU/g)	Females with Pups Infected/Total	Infection (log_10_ CFU/g)
*B. melitensis* 16M	0.62 ± 0.18 ^a^	4.47 ± 0.38 ^a^	4/4 ^a^	7.71 ± 1.82 ^a^	4/4 ^a^	6.37 ± 0.86 ^a^
*B. melitensis* Rev1	0.82 ± 0.44 ^a^	4.52 ± 1.01 ^a^	4/4 ^a^	8.17 ± 0.18 ^a^	4/4 ^a^	7.75 ± 0.54 ^a^
*B. suis* bv2	0.30 ± 0.08 ^ab^	6.26 ± 0.22 ^ab^	5/5 ^a^	4.27 ± 2.06 ^a^^b^	1/5 ^b^	2.05 ± 1.18 ^b^
Control	0.18 ± 0.04	1.52 ± 0	0/4	1.52 ± 0	0/4	1.52 ± 0

Pregnant CD1 mice were inoculated intraperitoneally at 4.5 DG with 6–7 × 10^5^ CFU/mouse of *Brucella* or 0.1 mL of PBS (control); 14 days later, all mice were necropsied and spleens, placentas and foetuses were aseptically removed and individually homogenised in 1:10 PBS. The number CFU/gram of tissue was determined by serial 10-fold dilutions and plating (detection limit: 33 CFU/g tissue = 1.52 log_10_). Fisher’s PLSD or Chi-square tests: *p* ≤ 0.05 ^a^ vs. PBS and ^b^ vs. *B. melitensis* groups.

**Table 2:** Reproductive and bacteriological parameters in mice at 18.5 DG.

Group	Mouse Number	Litter			Pups Infection	
		Size (No. Pups/Mouse)	Weight (g)	Viability ^&^ (%)	Females with Infected Pups/Total	Log_10_ CFU/g ^#^
*B. melitensis* 16M	1	Foetal reabsorption	/	0	/	5.25 ^†^
	2	Foetal reabsorption	/	0	/	4.46 ^†^
	3	18	6.7	0	+	5.34
	4	16	12.4	0	+	3.28
	5	11	12.1	0	+	3
mean ± SD		15.0 ± 3.6	10.4 ± 3.2	0 ^a^	3/3 ^a^	3.87 ± 1.28 ^a^
*B. melitensis* Rev1	1	11	11.5	72.2	-	1.52
	2	4	3.5	75	-	1.52
	3	11	3.1	0	+	3.45
	4	10	9.5	30	+	2.30
	5	9	8.0	0	+	4.28
mean ± SD		9.0 ± 2.6 ^a^	7.1 ± 3.3	35.4 ± 33.0 ^a^	3/5 ^a^	2.61 ± 1.09
*B. suis* bv2	1	11	6.2	81.8	-	1.52
	2	10	5.4	50	+	5.16
	3	9	7.0	100	-	1.52
	4	14	5.2	100	-	1.52
	5	10	6.6	100	-	1.52
mean ± SD		10.8 ± 1.7	6.1 ± 0.7	86.4 ± 19.5 ^b^	1/5 ^b^	2.25 ± 1.46
Control	1	13	7.7	100	-	1.52
	2	14	7.3	92.8	-	1.52
	3	13	7.1	100	-	1.52
mean ± SD		13.3 ± 0.6	7.4 ± 0.3	97.6 ± 4.2	0/3	1.52

Pregnant mice were inoculated and sampled as detailed in the footnote of Figure 3; ^&^ Percentage of viable pups/female, based on presence/absence of movement, skin colour, and body development; ^†^ Uterus infection. ^#^ Detection limit: 1.52 log_10_ CFU/g. Fisher’s PLSD and Chi-square tests: *p* ≤ 0.05 ^a^ vs. PBS and ^b^ vs. *B. melitensis*.

## Data Availability

The data presented in this study are available on request from the corresponding author.
